# The Profile and Content of Polyphenols and Carotenoids in Local and Commercial Sweet Cherry Fruits (*Prunus avium* L.) and Their Antioxidant Activity In Vitro

**DOI:** 10.3390/antiox8110534

**Published:** 2019-11-08

**Authors:** Dominika Średnicka-Tober, Alicja Ponder, Ewelina Hallmann, Agnieszka Głowacka, Elżbieta Rozpara

**Affiliations:** 1 Institute of Human Nutrition Sciences, Department of Functional and Organic Food, Warsaw University of Life Sciences, Nowoursynowska 159c, 02-776 Warsaw, Poland; dominika_srednicka_tober@sggw.pl (D.Ś.-T.); alicja_ponder@sggw.pl (A.P.); 2 Research Institute of Horticulture, Konstytucji 3 Maja 1, 96-100 Skierniewice, Poland; agnieszka.glowacka@inhort.pl (A.G.); elzbieta.rozpara@inhort.pl (E.R.)

**Keywords:** bioactive compounds, polyphenols, antioxidant activity, *Prunus avium* L., sweet cherries

## Abstract

The aim of this study was to evaluate and compare the content of a number of bioactive compounds and antioxidant activity of fruits of selected local and commercial sweet cherry (*Prunus avium* L.) cultivars. The experiment showed that the selected cultivars of sweet cherries differ significantly in the content of polyphenolic compounds and carotenoids. The fruits of commercial sweet cherry cultivars were, on average, richer in polyphenols (the sum of phenolic compounds determined chromatographically), flavonoids, as well as anthocyanins and were characterized by higher antioxidant activity when compared to the local, traditional cultivars. In the group of the traditional sweet cherry cultivars, particular attention could be paid to Black Late cv., showing the highest antioxidant activity of fruits. In the group of commercial sweet cherry cultivars, Cordia and Sylvia fruits could be recognized as being rich in bioactive compounds with high antioxidant activity. Yellow skin cultivars were characterized by the highest concentrations of carotenoids. Strong positive correlations between the identified bioactive compounds and antioxidant activity of fruits were also found. Although different cultivars of sweet cherries show a high variability in phenolics and carotenoids profiles as well as in the antioxidant activity of fruits, they all should be, similarly to other types of cherries, recognized as a rich source of bioactive compounds with an antioxidant potential.

## 1. Introduction

An increasing incidence of non-communicable diseases, including atherosclerosis, diabetes, and cancers, all closely associated with oxidative stress, motivates scientists to look for natural methods of disease prevention. Vegetables and fruit, including sweet cherries, represent potent sources of bioactive compounds, with strong health-promoting and disease-preventive activities [1,2,3,4,5]. These compounds present a strong antioxidant capacity and an ability to protect cell structures against oxidative damage, i.e., through the modulation of the antioxidant response, e.g., by increasing antioxidant enzyme activity, and the protection of mitochondrial functionality [4].

In 2016, global production of cherries reached 2.25 million tons. In Europe, Turkey is considered the largest producer of sweet cherries, with nearly 85 thousand hectares of sweet cherry orchards. Poland, with 9 thousand hectares, reached the fourth position. In both countries, vast majority of sweet cherries come from conventional horticultural production. In the organic system, where the range of available effective plant protection methods and treatments is limited, the choice of cultivar is an important aspect [2,3,6]. Sweet cherry cultivars differ not only in their resistance to diseases and performance in different environmental conditions, but also in their appearance (especially fruit color), fruit taste, as well as the content of biologically active compounds [7,8,9,10]. Some cultivars are especially recommended for organic production [11], while others are more popular in the conventional orchards. There is a large group of local, traditional sweet cherry cultivars, less popular than the commercial ones. To increase biodiversity in horticultural production, the protection and popularization of traditional and local sweet cherry cultivars is recommended.

The aim of this study was to compare the profile of a number of bioactive compounds (polyphenols and carotenoids) and antioxidant activity in the fruit of selected local and commercial sweet cherry cultivars (*Prunus avium* L.).

## 2. Materials and Methods

### 2.1. Plant Production

The experiment was conducted in the years 2012–2013. Nine cultivars of sweet cherries from the collection of the Research Institute of Horticulture in Skierniewice (Skierniewice, Poland) were selected for analysis. The identification of the cultivars in the collection was carried out according to the UPOV (International Union for the Protection of New Varieties of Plants) descriptors of the fruit shape, color, taste, firmness of the flesh, shape of the stone, characteristics of the stalk, and other morphological features of the tree, leaves, and flowers. To confirm the cultivars identification, genetic identity analysis was performed, using PCR-based methods [12,13]. Main characteristics of the selected cultivars are presented in Table 1.

In the experimental orchard, eight trees were located in one plot. The space between the trees was 3–4 × 4–4.5 m in a row. During the cultivation, all the standards for fertilization and protection of sweet cherries were applied. For experimental purposes, four from eight trees were used. From each tree, 1 kg of fully ripe fruits were collected. Time of harvest was 5 July (2012) and 7 July (2013).

### 2.2. Fruits Material Preparation

Fresh fruits were delivered to the laboratory, washed, and dried. Seeds from fruits were removed. Fruits were freeze-dried in a LabconCo freeze-drier (Kansas City, MI, USA) at −40 °C and under a pressure of 0.100 mbar. Freeze-dried fruit material was ground in an A-11 lab Mill and further stored at −80 °C before further chemical composition analyses.

### 2.3. Determination of Fruits Dry Matter

Dry matter content of fruit was determined before freeze drying. Fruit flesh was crushed with a Unipan blender. One gram of fruit pulp was dried using a KC-65 dryer, at a temperature of 105 °C, for 24 h. The dried samples were cooled and weighed. The content of dry matter was calculated in g/100 g, following the description in the Polish Norm PN-R-04013:1988 [14].

### 2.4. Determination of Polyphenols

Phenolics concentrations in fruit samples were analyzed using the method described by Hallmann et al. One hundred milligrams of freeze-dried sample were mixed in a plastic test tube with 1 mL of 80% methanol (by vortexing) and incubated for 15 min at 30 °C in an ultrasonic bath. The samples were then centrifuged (3780*× g* and 41,574*× g*). Five hundred microliters of extract was used for further HPLC analysis. The HPLC set-up included two LC-20AD pumps (Shimadzu, USA Manufacturing Inc., Canby, OR, USA), an SIL-20AC autosampler (Shimadzu, USA Manufacturing Inc., Canby, OR, USA), a CMB-20A system controller (Shimadzu, USA Manufacturing Inc., Canby, OR, USA), an SPD-20AV detector (Shimadzu, USA Manufacturing Inc., Canby, OR, USA), a CTO-20AC column oven (Shimadzu, USA Manufacturing Inc., Canby, OR, USA), and the Synergi Fusion-RP 80i column (250 × 4.60 mm) (Phenomenex Inc., Torrance, CA, OR, USA). A gradient programme with two mobile phases was used: an aqueous solution of 10% (v/v) acetonitrile (phase A) and 55% (v/v) acetonitrile (phase B), both acidified to pH 3.0 by ortho-phosphoric acid. The programme was as follows: 0–21 min—95% Solvent A and 5% Solvent B; 22–25 min—50% Solvent A and 50 % Solvent B; 26–27 min—20% Solvent A and 80% Solvent B; 28–32 min—20% Solvent A and 80 % Solvent B; 32–36 min—95% Solvent A and 5% Solvent B. The time of the analysis was 36 min, with a flow rate of 1 mL min^−1^, and a wavelength range of 250–370 nm. Fluka and Sigma-Aldrich (Poznan, Poland) external standards were used for the identification of polyphenols [15].

### 2.5. Determination of Carotenoids

Carotenoid compounds were determined with the method described by Hallmann et al. [15]. A weighed amount of freeze-dried fruit sample (100 mg) was put into a plastic test tube, and 5 mL of pure acetone and 1 mg magnesium carbonate were then added. The solution was mixed thoroughly by vortexing and incubated in an ultrasonic bath (10 min at 0 °C). The samples were then centrifuged at 3800*× g* and re-centrifuged at 42,685*× g*. An aliquot of 900 μL of extract was taken for analysis by HPLC. To determine carotenoids, the HPLC set-up (Shimadzu, USA Manufacturing Inc., OR, Canby, USA) consisted of two LC-20AD pumps, a CMB-20A system controller, an SIL-20AC autosampler, an ultraviolet–visible SPD-20AV detector, and a CTD-20AC oven. A Synergi Max-RP 80i column (250 × 4.60 mm) (Phenomenex Inc., Torrance, CA, USA) was used, and elution was by using gradient flow with two mobile phases: (A) acetonitrile/methanol (90:10) and (B) methanol/ethyl acetate (64:36). The used gradient program was: 0–16 min—95% Solvent A and 5% Solvent B; 17–21 min—50% Solvent A and 50% Solvent B; 22–25 min—20% Solvent A and 80% Solvent B; 26–28 min—20% Solvent A and 80% Solvent B. The analysis time was 28 min, the flow rate was 1 mL min^−1^, and the wavelength range for detection was 445–450 nm. Carotenoids were identified based on Fluka and Sigma–Aldrich (Poznan, Poland) external standards [15].

### 2.6. Determination of Anthocyanins

The first step of anthocyanin extraction (in 80% of ethanol) was as described for polyphenols. Next, 2.5 mL of supernatant was put in a plastic tube, and 2.5 mL of 10 M HCl and 5.0 mL 100% methanol were added. The samples were cooled at 5 °C for 10 min. One milliliter of extract was used in HPLC analysis. The elution of anthocyanins was produced by using an isocratic flow with one mobile phase: 5% acetic acid, acetonitrile, and methanol (70:10:20) v/v/v. The flow rate was 1.5 mL min^−1^, and the wavelength for detection was 530 nm. Anthocyanins were identified based on Sigma-Aldrich (Poznan, Poland) external standards (cyanidine-3.5-di-O-rutinoside and cyanidine-3.5-di-O-glucoside) with a purity of 99.9% [16].

### 2.7. Determination of Vitamin C

l-ascorbic acid was determined by the HPLC method, as previously described by Rekha et al. [17]. The freeze-dried sample was weighed (100 mg) and put into a plastic tube. Next, 5% *metha*-phosphoric acid was added. The sample was incubated for 15 min at 30 °C in an ultrasonic bath and centrifuged at a speed of 3780*× g*. One milliliter of supernatant was collected and re-centrifuged at 41,574*× g*. Next, 900 μL of supernatant were used in the HPLC analysis. Identification of l-ascorbic acid was based on Sigma-Aldrich (Poznan, Poland) external standard with a purity of 99.9%. The analysis was performed using a Synergi Hydro-RP 80i column (250 × 4.60 mm) (Phenomenex Inc., Torrance, CA, USA), with one mobile phase: phosphate buffer 4.0. The wavelength for detection was 280 nm, and the time of the analysis was 10 min [17].

### 2.8. Determination of Antioxidant Activity (AA)

Antioxidant activity of the samples was measured with a method previously described by Re et al. [18]. Two hundred fifty milligrams of the freeze-dried sweet cherry powder were weighed into a plastic tube with 25 mL of distilled water, shaken with a vortex shaker (1 min, 2000 rpm), and incubated (IKA KS 4000 incubator, 45 min, 30 °C, 200 rpm) (IKA® Poland Sp. z o.o., Warsaw, Poland). The sample was then shaken again on a vortex shaker (40 s) and centrifuged (Centrifuge MPW-380R, 20 min, 2 °C, 7500 rpm) (MPW MED. INSTRUMENTS, Warsaw, Poland). The supernatant was collected for antioxidant activity analysis. Extract solution was measured with 3.0 mL of an ABTS•^+^ cationic solution in phosphate-buffered saline. Absorbance was measured after 6 min of incubation at room temperature, at a wavelength of 734 nm, using a Helios γ spectrophotometer (Spectro-Lab, Warsaw, Poland). Results were expressed in mmol of TE (Trolox equivalents) per 100 g fresh weight of fruits [18].

### 2.9. Statistical Analysis

Data were analyzed using one-way analysis of variance (ANOVA) followed by Duncan post hoc test (α = 0.05) to compare (a) individual sweet cherry cultivars and (b) a group of local cultivars vs. a group of commercial cultivars. The results are expressed as means and standard deviations for the individual cultivars and groups. Concentrations of the analyzed compounds were expressed per 100 g of fresh weight of fruits. Pearson Product-Moment Correlation analyses between the studied phytochemicals concentrations and the fruit antioxidant activity were additionally undertaken. All statistical analyses were performed using Statgraphics 15.2.11.0 software (StatPoint Technologies, Inc, Warrenton, VI, USA).

## 3. Results

The results on the concentrations of dry matter, vitamin C, and polyphenols (sums of the compounds determined chromatographically) in sweet cherry fruit harvested in 2012 are presented in Table 2. The analysis indicated that the cultivar has a significant influence (*p* < 0.0001) on dry matter content in the fruit. The highest dry matter content was found in the cultivar Elton Heart. The cultivars Black from Turwia and Drogans Gelbe Knorpelkirsche were characterized by the lowest content of dry matter. The differences in the content of dry matter between the groups of local vs. commercial cultivars were not statistically significant. The content of polyphenolic compounds in individual cultivars of sweet cherries differed significantly. The Kordia cv., Black Late cv., and Sylvia cv. were characterized by the highest contents of polyphenols (sum), while Black Late cv. was richest in phenolic acids. The highest contents of flavonoids (sum) as well as anthocyanins were found in the Kordia and Sylvia cultivars. Fruits of Sylvia and Black Late cv. were characterized by the highest antioxidant activity. The differences in the content of polyphenols (*p* = 0.0041) and flavonoids (*p* < 0.0001) as well as antioxidant activity of fruits (*p* < 0.0001) between the groups of local and commercial cultivars were also statistically significant. On average, fruits of commercial cultivars were characterized by a higher content of polyphenols (sum) and flavonoids and higher antioxidant activity compared to the fruits of local cultivars. It was observed that the content of individual polyphenolic compounds differed significantly between the sweet cherry fruits of different cultivars (Table 3). In 2012, Elton Heart cv. was characterized by the highest level of quercetin derivates and kaempferol content compared to the rest of the examined cultivars. Two anthocyanins were identified in the sweet cherry fruits. Fruits belonging to the commercial cultivars were characterized by significantly higher concentrations of cyanidin-3,5-di-O-rutinoside (*p* < 0.0001) compared to the local cultivars. It is worth paying attention to the local cherry cultivars with fruits with a dark skin color, such as Black Late and Black from Turwia. Fruits of these cultivars contained significantly more cyanidin-3,5-di-O-rutinoside compared to Altenburger Melonenkirsche, Bütners Rote Knorpelkirsche, and Elton Heart. At the same time, anthocyanins were not detected in the fruits of Drogans Gelbe Knorpelkirsche cv.

The content of dry matter, vitamin C, and polyphenols (sum) in sweet cherry fruit harvested in 2013 is shown in Table 4. Fruits of Elton Heart cv. were characterized by the highest dry mater content. The highest content of vitamin C was found in fruits of Bütners Rote Knorpelkirsche cv., while the lowest content of this compound was found in Drogans Gelbe Knorpelkirsche and Elton Heart cv. fruits. The Sylvia cv. was characterized by the highest content of polyphenols (*p* < 0.0001) and phenolic acids (*p* < 0.0001) and the highest antioxidant activity (*p* < 0.0001). The highest concentrations of the identified flavonoids (sum) (*p* < 0.0001) and anthocyanins (*p* < 0.0001) were found in the fruits of Kordia and Sylvia cv. The highest content of flavonols and flavones was found in the fruits of Bütners Rote Knorpelkirsche cv. The fruits of the commercial sweet cherry cultivars were generally richer in vitamin C and the identified polyphenolic compounds (polyphenols, flavonoids, and anthocyanins) and were characterized by higher antioxidant activity when compared to the local cultivars (*p* < 0.0001). The highest concentrations of individual polyphenolic compounds such as caffeic acid, quercetin-3-*O*-rutinoside, and kaempferol were found in the fruits of Regina cv. (Table 5). The highest contents of cyanidin-3-*O*-rutinoside were found in Kordia and Sylvia cv. Sylvia cv. was also characterized by the highest concentrations of gallic acid. The Bütners Rote Knorpelkirsche cv. fruits contained the highest concentrations of luteolin, while fruits of Elton Heart cv. were characterized by the highest concentrations of quercetin-3-*O*-glucoside. Differences between the new commercial and the traditional cultivars in the content of individual polyphenolic compounds were statistically significant only in the case of caffeic acid (*p* = 0.009), luteolin (*p* = 0.031), and cyanidin-3-*O*-rutinoside (*p* < 0.0001).

In both years of the experiment, carotenoids were detected only in the fruit of local cherry cultivars. Fruits with yellow skin (Drogans Gelbe Knorpelkirsche cv. and Elton Heart cv.) were characterized by the highest concentrations of all detected carotenoids (Table 2, Table 4, and Table 6).

Correlation analyses between the concentrations of the studied phytochemicals (phenolic compounds and carotenoids) and antioxidant activity of fruits were additionally performed (Table 7). The strongest positive correlations between fruit phenolics concentrations and antioxidant activity were found for local sweet cherry cultivars such as Altenburger Melonenkirsche (*r^2^* = 0.9017, *p* = 0.0001), Elton Heart (*r^2^* = 0.8923, *p* = 0.0001), Drogans Gelbe Knorpelkirsche (*r^2^* = 0.8873, *p* = 0.0001), Black Late (r^2^= 0.8400, *p* = 0.0005), and Black from Turwia (*r^2^* = 0.8249, *p* = 0.0007). Weaker, but still significant correlation between antioxidant activity and phenolics concentrations in fruits were observed for Bütners Rote Knorpelkirsche cv. (*r^2^* = 0.7593, *p* = 0.0022) (Table 7). Similar strong correlations were identified for all three commercial cultivars: Cordia (*r^2^* = 0.9315, *p* < 0.0001), Regina (*r^2^* = 0.8272, *p* = 0.0007), and Sylvia (*r^2^* = 0.8054, *p* = 0.001) (Table 7). Moreover, in case of the fruits with yellow skin (Drogans Gelbe Knorpelkirsche cv. and Elton Heart cv.), antioxidant activity was positively associated with the concentrations of β-carotene in fruits (Table 7).

## 4. Discussion

The presented study indicates that individual cultivars of sweet cherries differ from each other in the content of polyphenolic compounds and carotenoids. Phenolics such as flavonoids, and particularly anthocyanins, are responsible for the red-purple color of the fruit. That is why fruits of sweet cherry cultivars with a deep purple color tested in the presented study contained more anthocyanins compared to the other cultivars. Fruits with yellow skin and a pink blush on the small surface contained less anthocyanins. Similar results were observed by Usenik et al., who reported that Ferrador cv., which was characterized by bi-color fruits (mostly yellow with small pink blushes), contained lower levels of anthocyanins (1.15 mg CGE g^−1^ FW) compared to deep purple cultivars [9]. Demir reported two cultivars of sweet cherry (Napoleon cv and Lambert cv.) with yellow skin and flesh to be characterized by higher levels of carotenoids (β-carotene, α-carotene, and xanthophylls) compared to fruits with a purple color [19].

Chaovanalikit et al. also looked into the concentrations of bioactive compounds in fruits of several sweet cherry cultivars [20]. They found the highest contents of phenolics in fruit skin of Bing cv., while Royal Ann cv. was characterized by the highest content of hydroxycinnamates. On the other hand, Montmorency cv. contained the highest concentrations of epicatechin, procyanidins, and flavonol glycosides. Fruits of Drogans Gelbe Knorpelkirsche cv., characterized by a yellow skin and flesh color, did not contain anthocyanins. Mozetic et al. also analyzed the profile of phenolic compounds in sweet cherry fruits. They identified compounds such as chlorogenic acid, 3’-*p*-coumaroylquinic acid, cyanidin-3-*O*-glucoside, cyanidin-3-*O*-rutinoside, peonidin-3-*O*-rutinoside, peonidin-3-*O*-glucoside, and pelargonidin-3-*O*-rutinoside in the fruit [21].

Kim et al. [22] analyzed concentrations of phenolics and their anti-neurodegenerative activities in four sweet cherry and four sour cherry cultivars. The concentrations of phenolics in their study ranged from 92.1–146.8 mg GE 100 g^−1^ FW in sweet cherries to 146.1–312.4 mg GE 100 g^−1^ FW in sour cherries. At the same time, concentrations of anthocyanins in sweet and sour cherry fruits ranged from 30.2 to 76.6 and from 49.1 to 109.2 mg CGE 100 g^−1^ FW, respectively. The highest concentrations of phenolics, including anthocyanins, were identified in sweet cherries of Hartlan cv. Cyanidin, and peonidin derivatives appeared to be predominant phenolics in the cherry fruits. The identified hydroxycinnamic acids consisted of chlorogenic acid, neochlorogenic acid, and *p*-coumaric acid derivatives. Glycosides of kaempferol, quercetin, and isorhamnetin were also identified. The authors concluded that sour cherries are generally richer in phenolic compounds, and especially anthocyanins and hydroxycinnamic acids, compared to sweet cherry fruits.

The variation in the content of phenolic compounds in fruits of different sweet cherry cultivars were also demonstrated by Jakobek at al. [23]. Major phenolic acids identified in sweet cherries in this study included neochlorogenic acid (1.8–5.0 mg 100 g^−1^ FW), chlorogenic acid (1.9–6.2 mg 100 g^−1^ FW), and *p*-coumaric acid derivatives (1.5–12.5 mg 100 g**^−1^** FW). The amount of flavonol quercetin-3-*O*-rutinoside (0.8–3.7 mg 100 g^−1^ FW) was significant as well.

Prvulovic et al. measured the concentrations of polyphenols (total), tannins, and flavonoids as well as the antioxidant capacity in fruits of selected sweet cherry genotypes. They reported polyphenol concentrations in a range from 4.12 to 8.34 mg GAE/g dry weight of fruits, flavonoid concentrations of 0.42−1.56 mg of rutin equivalents/g dry fruit, and total anthocyanin content reaching between 0.35 and 0.69 mg cyanidin 3-glucoside equivalent/g dry fruit weight [24].

Phenolic compounds are strong antioxidants. This was confirmed in our study by a significant positive correlation between polyphenol content in fruits and fruit antioxidant activity (Table 7). It is well proven that such compounds protect cell structures against oxidative damage, i.e., through the modulation of the antioxidant response, e.g., by increasing antioxidant enzyme activity, and the protection of mitochondrial functionality [4,5].

Sweet cherries with a deep purple color (rich in anthocyanins) showed high antioxidant activity, which confirms the antioxidant potential of these bioactive compounds [25]. However, cultivars with yellow skin and flesh (without or with low concentrations of anthocyanins) also showed significant antioxidant potential, correlated with the concentrations of another group of strong antioxidants—carotenoids—in the fruits.

## 5. Conclusions

The experiment showed that the cultivars of sweet cherries differ significantly in the content of polyphenolic compounds. The fruits of commercial sweet cherry cultivars were, on average, richer in polyphenols (sum of the identified compounds), flavonoids (sum), as well as anthocyanins (sum) and were characterized by a higher antioxidant activity when compared to the local, traditional cultivars. This was confirmed in both years of the experiment. In the group of the traditional sweet cherry cultivars, particular attention could be paid to Black Late cv., showing the highest antioxidant activity of fruits. In the group of commercial sweet cherry cultivars, Cordia and Sylvia fruits could be recognized as being rich in bioactive compounds and characterized by high antioxidant activity. Strong positive correlations between fruit phenolic concentrations and the fruit antioxidant activity were also identified. Although different cultivars of sweet cherries show a high variability in phenolics and carotenoids profiles as well as in antioxidant activity of fruits, they all should be, similarly to other types of cherries, recognized as a rich source of bioactive compounds with an antioxidant, thus health-promoting and disease-preventive, potential.

## Figures and Tables

**Table 1:** Characterization of sweet cherry varieties.

Sweet Cherry Cultivar	Year of Origin	Country of Origin	Fruits Characterization	S-Allele Composition
Altenburger Melonenkirsche	1795	Germany	Fruit skin is glossy, yellow with small red blush; fruits are round to partially elliptic; flesh is yellow.	S_3_S_4_
Bütners Rote Knorpelkirsche	1845	Germany	Fruit skin is glossy, yellow with a large red blush; fruits are round; flesh is yellow to pink.	S_3_S_4_
Black from Turwia	1850	Poland	Fruit skin is glossy, dark red, black when fully mature; fruits are large or very large, round; flesh is dark red.	-
Black Late	1924	United Kingdom	Fruit skin is glossy, dark red, black when fully mature; fruits are large or very large, round; flesh is dark red.	S_3_S_4_
Drogans Gelbe Knorpelkirsche	1850	Hungary	Fruit skin is yellow, matte; fruits are small and round; flesh is slightly creamy to yellow.	S_1_S_5_
Elton Heart	1806	United Kingdom	Fruit skin is yellow, matte; fruits are small, hearts-shaped; flesh is slightly yellow.	S_3_S_6_
Kordia	1991	Czech Republic	Fruit skin is dark red, glossy; fruits are very big, hearts-shaped; flesh is deep purple.	S_3_S_6_
Regina	1950	Germany	Fruit skin is dark red, glossy; fruits are very big, round; flesh is deep purple.	S_1_S_3_
Sylvia	1950	Canada	Fruit skin is dark red, glossy; fruits are very big, round; flesh is deep purple.	S_1_S_4_

**Table 2:** The content of dry matter (in g 100 g^−1^ FW), vitamin C, polyphenols, carotenoids (in mg 100 g^−1^ FW) and antioxidant activity (mmol Trolox 100 g^−1^ FW) in sweet cherry fruit harvested in 2012.

Sweet Cherry Cultivars	Dry Matter	Vitamin C	Polyphenols (sum) ^3^	Phenolic Acids (sum) ^3^	Flavonoids (sum) ^3^	Flavonols & Flavones (sum) ^3^	Anthocyanins (sum) ^3^	Carotenoids (sum) ^3^	Antioxidant Activity
Local Cultivars									
Altenburger Melonenkirsche	20.90 ± 0.04 ^e,1,2^	40.45 ± 1.72 ^d^	62.43 ± 0.52 ^b^	38.81 ± 0.16 ^c,d^	23.62 ± 0.61 ^b^	5.32 ± 0.03 ^c,d^	18.30 ± 0.58 ^b^	3.56 ± 0.05 ^a^	220.9 ± 1.93 ^c^
Bütners Rote Knorpelkirsche	22.68 ± 0.05 ^f^	42.89 ± 1.99 ^d^	71.68 ± 2.02 ^c^	40.01 ± 0.29 ^d^	31.68 ± 1.78 ^c^	6.48 ± 0.11 ^e^	25.19 ± 1.74 ^c^	3.92 ± 0.05 ^b^	308.7 ± 5.72 ^d^
Black from Turwia	16.25 ± 0.04 ^a^	33.27 ± 0.52 ^c^	104.74 ± 2.68 ^d^	40.28 ± 0.21 ^d^	64.46 ± 2.48 ^d^	5.30 ± 0.06 ^c,d^	59.15 ± 2.42 ^d^	N.D.	489.3 ± 4.63 ^e^
Black Late	19.68 ± 0.75 ^d^	42.76 ± 1.84 ^d^	141.61 ± 4.11 ^g^	80.50 ± 3.02 ^f^	61.11 ± 1.53 ^d^	5.36 ± 0.20 ^d^	55.75 ± 1.54 ^d^	N.D.	632.3 ± 6.12 ^g^
Drogans Gelbe Knorpelkirsche	16.24 ± 0.02 ^a^	17.29 ± 0.15 ^a^	41.51 ± 0.25 ^a^	36.78 ± 0.22 ^b,c^	4.72 ± 0.07 ^a^	4.72 ± 0.07 ^b^	N.D.	7.02 ± 0.06 ^c^	128.9 ± 3.38 ^a^
Elton Heart	23.28 ± 0.04 ^g^	22.88 ± 0.41 ^b^	47.88 ± 0.26 ^a^	29.37 ± 0.07 ^a^	18.51 ± 0.20 ^b^	6.42 ± 0.08 ^e^	12.09 ± 0.12 ^b^	9.02 ± 0.13 ^d^	178.9 ± 4.61 ^b^
**Commercial Cultivars**									
Kordia	17.29 ± 0.05 ^b^	33.07 ± 0.27 ^c^	145.77 ± 4.21 ^g^	49.69 ± 0.18 ^e^	96.08 ± 4.06 ^f^	4.47 ± 0.11 ^a^	91.61 ± 3.94 ^f^	N.D.	590.8 ± 2.29 ^f^
Regina	18.96 ± 0.08 ^c^	34.44 ± 0.31 ^c^	114.67 ± 4.57 ^e^	35.60 ± 0.06 ^b,c^	79.06 ± 4.50 ^e^	5.06 ± 0.10 ^c^	74.00 ± 4.60 ^e^	N.D.	306.9 ± 2.02 ^d^
Sylvia	18.95 ± 0.09 ^c^	32.59 ± 0.17 ^c^	133.28 ± 6.05 ^f^	39.35 ± 0.23 ^d^	93.93 ± 5.96 ^f^	5.31 ± 0.16 ^c,d^	88.63 ± 5.87 ^f^	N.D.	692.9 ± 5.88 ^g^
local cultivars av.	19.84 ± 2.81 ^a^	33.25 ± 10.07 ^a^	78.31 ± 34.91 ^a^	44.29 ± 16.65 ^a^	34.02 ± 21.93 ^a^	5.60 ± 0.65 ^a^	28.41 ± 21.95 ^a^	5.90 ± 2.28	326.5 ± 79.1
commercial cultivars av.	18.40 ± 0.79 ^a^	33.37 ± 0.83 ^a^	131.24 ± 13.73 ^b^	41.55 ± 5.96 ^a^	89.69 ± 9.02 ^b^	4.94 ± 0.38 ^a^	84.75 ± 9.11 ^b^	N.D.	530.2 ± 63.4
***p*-value: group of cultivars**	N.S.	N.S.	0.0041	N.S.	<0.0001	N.S.	<0.0001	-	<0.0001
***p*-value: cultivar**	<0.0001	<0.0001	<0.0001	<0.0001	<0.0001	<0.0001	<0.0001	<0.0001	<0.0001

^1^ Data are presented as mean ± SD, with ANOVA *p*-values (*n* = 4); ^2^ Means in the same columns followed by the same letter (^a–h^) are not significantly different at the 5% level of probability (*p* < 0.05); ^3^ Sum of phenolics determined chromatographically within the study; N.S.: not significant; N.D.: not detected.

**Table 3:** The content of individual phenolic compounds (in mg 100 g^−1^ FW) in sweet cherry fruit harvested in 2012.

Sweet Cherry Cultivars	Gallic Acid	Chlorogenic Acid	Caffeic Acid	Quercetin-3-*O*-Rutinoside	Quercetin-3-*O*-Glucoside	Luteolin	Kaempferol	Cyanidin-3-*O*-Rutinoside	Cyanidin-3-*O*-Glucoside
Local Cultivars									
Altenburger Melonenkirsche	33.91 ± 0.08 ^a,b,1,2^	3.07 ± 0.04 ^e^	1.82 ± 0.06 ^g^	0.89 ± 0.01 ^a^	1.19 ± 0.05 ^c^	1.78 ± 0.08 ^f^	1.46 ± 0.01 ^a,b^	14.60 ± 0.53 ^b^	3.67 ± 0.05 ^d,e^
Bütners Rote Knorpelkirsche	35.35 ± 0.04 ^b,c^	3.43 ± 0.19 ^f^	1.23 ± 0.07 ^c,d^	1.05 ± 0.07 ^b^	1.24 ± 0.03 ^c,d^	2.54 ± 0.10 ^g^	1.65 ± 0.06 ^d^	22.24 ± 1.58 ^c^	2.95 ± 0.16 ^b^
Black from Turwia	38.30 ± 0.16 ^d^	0.58 ± 0.01 ^b^	1.39 ± 0.06 ^e^	1.31 ± 0.01 ^e^	0.91 ± 0.08 ^b^	1.43 ± 0.08 ^c,d^	1.65 ± 0.01 ^d^	55.63 ± 2.28 ^d^	3.52 ± 0.15 ^c,d^
Black Late	77.03 ± 2.92 ^f^	2.34 ± 0.08 ^d^	1.13 ± 0.02 ^b,c^	1.23 ± 0.09 ^d,e^	1.16 ± 0.05 ^c^	1.27 ± 0.02 ^b^	1.70 ± 0.09 ^d^	51.91 ± 1.54 ^d^	3.84 ± 0.08 ^d,e^
Drogans Gelbe Knorpelkirsche	33.89 ± 0.01 ^b^	1.96 ± 0.16 ^c^	0.92 ± 0.05 ^a^	1.05 ± 0.02 ^b^	0.90 ± 0.03 ^b^	1.35 ± 0.05 ^b,c^	1.43 ± 0.01 ^a^	N.D.	N.D.
Elton Heart	24.45 ± 0.05 ^a^	3.37 ± 0.03 ^f^	1.54 ± 0.03 ^f^	1.42 ± 0.01 ^f^	1.36 ± 0.06 ^d^	1.67 ± 0.02 ^e,f^	1.98 ± 0.01 ^e^	8.88 ± 0.15 ^b^	3.21 ± 0.04 ^b,c^
**Commercial Cultivars**									
Kordia	46.69 ± 0.16 ^e^	1.84 ± 0.13 ^c^	1.16 ± 0.01 ^b,c,d^	1.12 ± 0.04 ^b,c^	0.70 ± 0.04 ^a^	1.11 ± 0.08 ^a^	1.53 ± 0.03 ^b,c^	87.86 ± 3.72 ^f^	3.75 ± 0.23 ^d,e^
Regina	33.99 ± 0.03 ^b^	0.36 ± 0.04 ^a^	1.25 ± 0.02 ^d^	1.16 ± 0.05 ^c,d^	0.83 ± 0.05 ^a,b^	1.46 ± 0.03 ^c,d^	1.62 ± 0.04 ^c,d^	70.09 ± 4.52 ^e^	3.91 ± 0.08 ^e^
Sylvia	36.38 ± 0.20 ^c,d^	1.88 ± 0.03 ^c^	1.09 ± 0.03 ^b^	1.24 ± 0.04 ^d,e^	0.82 ± 0.10 ^a,b^	1.57 ± 0.06 ^d,e^	1.68 ± 0.03 ^d^	85.01 ± 5.53 ^f^	3.61 ± 0.33 ^d,e^
local cultivars av.	40.49 ± 16.93 ^a^	2.46 ± 1.00 ^b^	1.34 ± 0.30 ^a^	1.16 ± 0.19 ^a^	1.13 ± 0.18 ^b^	1.67 ± 0.43 ^a^	1.64 ± 0.19 ^a^	25.54 ± 21.1 ^a^	2.87 ± 1.32 ^a^
commercial cultivars av.	39.02 ± 5.51 ^a^	1.36 ± 0.71 ^a^	1.17 ± 0.07 ^a^	1.17 ± 0.06 ^a^	0.78 ± 0.09 ^a^	1.38 ± 0.21 ^a^	1.61 ± 0.07 ^a^	80.99 ± 9.08 ^b^	3.76 ± 0.27 ^a^
***p*-value: group of cultivars**	N.S.	0.047	N.S.	N.S.	0.0008	N.S.	N.S.	<0.0001	N.S.
***p*-value: cultivar**	<0.0001	<0.0001	<0.0001	<0.0001	<0.0001	<0.0001	<0.0001	<0.0001	<0.0001

^1^ Data are presented as mean ± SD, with ANOVA *p*-values (*n* = 4); ^2^ Means in the same columns followed by the same letter (^a–f^) are not significantly different at the 5% level of probability (*p* < 0.05); N.S.: not significant; N.D.: not detected..

**Table 4:** The content of dry matter (in g 100 g^−1^ FW), vitamin C, polyphenols, carotenoids (in mg 100 g^−1^ FW), and antioxidant activity (mmol Trolox 100 g^−1^ FW) in sweet cherry fruit harvested in 2013.

Sweet Cherry Cultivars	Dry Matter	Vitamin C	Polyphenols (sum)^3^	Phenolic Acids (sum)^3^	Flavonoids (sum)^3^	Flavonols & Flavones (sum)^3^	Anthocyanins (sum)^3^	Carotenoids (sum)^3^	Antioxidant Activity
Local Cultivars									
Altenburger Melonenkirsche	16.67 ± 0.13 ^c,d,1,2^	21.73 ± 1.23 ^b,c^	97.81 ± 2.97 ^d^	75.89 ± 2.26 ^e^	21.92 ± 0.71 ^b^	2.49 ± 0.09 ^b^	19.42 ± 0.80 ^b^	3.92 ± 0.04 ^a^	271.2 ± 14.8 ^c^
Bütners Rote Knorpelkirsche	16.03 ± 0.20 ^b,c^	25.09 ± 3.18 ^c^	80.76 ± 6.70 ^c^	47.53 ± 4.29 ^d^	33.23 ± 2.41 ^c^	5.33 ± 0.08 ^f^	27.89 ± 2.39 ^c^	4.32 ± 0.03 ^b^	351.5 ± 27.9 ^e^
Black from Turwia	16.27 ± 0.16 ^c,d^	16.12 ± 4.18 ^a,b^	97.10 ± 3.01 ^d^	37.63 ± 4.76 ^c^	60.36 ± 2.81 ^d^	4.75 ± 0.06 ^e^	55.61 ± 2.79 ^d^	N.D.	503.5 ± 6.0 ^f^
Black Late	17.31 ± 0.24 ^d,e^	18.22 ± 3.49 ^a,b^	87.88 ± 4.43 ^c^	26.50 ± 3.79 ^a^	61.38 ± 2.01 ^d^	4.01 ± 0.10 ^d^	57.37 ± 2.00 ^d^	N.D.	654.1 ± 8.9 ^h^
Drogans Gelbe Knorpelkirsche	15.99 ± 0.80 ^b,c^	13.94 ± 2.60 ^a^	34.39 ± 0.79 ^a^	32.32 ± 0.69 ^b^	2.07 ± 0.10 ^a^	2.07 ± 0.11 ^a^	N.D.	8.15 ± 0.05 ^c^	148.2 ± 9.0 ^a^
Elton Heart	18.03 ± 0.289 ^e^	13.02 ± 1.69 ^a^	49.10 ± 0.97 ^b^	33.58 ± 1.03 ^b,c^	15.52 ± 0.20 ^b^	2.23 ± 0.01 ^a^	13.28 ± 0.21 ^b^	10.780.17 ^d^	198.6 ± 5.5 ^b^
**Commercial Cultivars**									
Kordia	14.59 ± 1.56 ^a^	18.76 ± 6.83 ^a,b,c^	136.41 ± 5.33 ^f^	36.89 ± 0.28 ^b,c^	99.51 ± 5.19 ^f^	4.14 ± 0.35 ^d^	95.37 ± 4.10 ^f^	N.D.	602.8 ± 3.8 ^g^
Regina	17.88 ± 0.26 ^e^	21.68 ± 3.34 ^b,c^	126.49 ± 5.75 ^e^	45.20 ± 0.37 ^d^	81.29 ± 5.92 ^e^	4.62 ± 0.06 ^e^	76.66 ± 5.92 ^e^	N.D.	316.4 ± 4.2 ^e^
Sylvia	14.88 ± 0.59 ^a,b^	21.09 ± 1.78 ^b,c^	186.24 ± 10.19 ^g^	90.51 ± 2.63 ^f^	95.72 ± 7.56 ^f^	3.45 ± 0.15 ^c^	92.27 ± 7.43 ^f^	N.D.	726.3 ± 10.2 ^h^
local cultivars av.	16.72 ± 0.82 ^a^	18.02 ± 5.02 ^a^	74.66 ± 25.29 ^a^	42.24 ± 17.03 ^a^	32.41 ± 22.81 ^a^	3.48 ± 1.32 ^a^	28.93 ± 21.85 ^a^	6.79 ± 2.84	354.5 ± 76.5 ^a^
commercial cultivars av.	15.78 ± 1.79 ^a^	20.51 ± 4.13 ^a^	149.71 ± 28.46 ^b^	57.53 ± 25.03 ^a^	92.17 ± 9.96 ^b^	4.07 ± 0.55 ^a^	88.10 ± 10.21 ^b^	N.D.	548.5 ± 71.5 ^b^
***p*-value: group of cultivars**	N.S.	N.S.	<0.0001	N.S.	<0.0001	N.S.	<0.0001	-	<0.0001
***p*-value: cultivar**	<0.0001	0.008	<0.0001	<0.0001	<0.0001	<0.0001	<0.0001	<00001	<0.0001

^1^ Data are presented as mean ± SD, with ANOVA *p*-values (*n* = 4); ^2^ Means in the same columns followed by the same letter (^a–h^) are not significantly different at the 5% level of probability (*p* < 0.05); ^3^ Sum of phenolics determined chromatographically within the study; N.S.: not significant; N.D.: not detected.

**Table 5:** The content of individual phenolic compounds (in mg 100 g^−1^ FW) in sweet cherry fruit harvested in 2013.

Sweet Cherry Cultivars	Gallic Acid	Chlorogenic Acid	Caffeic Acid	Quercetin-3-*O*-Rutinoside	Quercetin-3-*O*-Glucoside	Luteolin	Kaempferol	Cyanidin-3-*O*-Rutinoside	Cyanidin-3-*O*-Glucoside
Local Cultivars									
Altenburger Melonenkirsche	60.76 ± 0.64 ^f,1,2^	14.53 ± 1.58 ^d^	0.59 ± 0.06 ^b^	0.65 ± 0.18 ^b^	0.35 ± 0.03 ^d^	1.17 ± 0.07 ^f^	0.32 ± 0.01 ^a,b^	17.09 ± 0.76 ^b^	2.33 ± 0.04 ^d,e^
Bütners Rote Knorpelkirsche	36.09 ± 2.16 ^e^	10.79 ± 2.16 ^a,b^	0.64 ± 0.01 ^b,c^	2.35 ± 0.08 ^d^	0.46 ± 0.01 ^e^	2.23 ± 0.11 ^h^	0.30 ± 0.01 ^a,b^	26.03 ± 2.26 ^c^	1.86 ± 0.12 ^b^
Black from Turwia	23.92 ± 4.56 ^b,c^	13.09 ± 0.22 ^c,d^	0.62 ± 0.01 ^b^	2.45 ± 0.02 ^d^	0.25 ± 0.01 ^b^	1.55 ± 0.01 ^g^	0.50 ± 0.06 ^d^	53.38 ± 2.68 ^d^	2.22 ± 0.12 ^c,d^
Black Late	14.72 ± 3.61 ^a^	11.04 ± 0.18 ^a,b^	0.74 ± 0.01 ^c^	2.37 ± 0.03 ^d^	0.28 ± 0.01 ^b,c^	0.84 ± 0.02 ^b^	0.52 ± 0.06 ^d^	54.95 ± 2.00 ^d^	2.42 ± 0.07 ^d,e^
Drogans Gelbe Knorpelkirsche	21.61 ± 0.13 ^b,c^	10.26 ± 0.55 ^a^	0.45 ± 0.02 ^a^	0.63 ± 0.03 ^b^	0.21 ± 0.01 ^a^	0.89 ± 0.04 ^b,c^	0.33 ± 0.02 ^a,b^	N.D.	N.D.
Elton Heart	22.92 ± 1.16 ^b,c^	10.21 ± 0.15 ^a^	0.45 ± 0.01 ^a^	0.24 ± 0.01 ^a^	0.53 ± 0.02 ^f^	1.10 ± 0.01 ^e,f^	0.36 ± 0.01 ^b^	11.26 ± 0.23 ^b^	2.03 ± 0.03 ^b,c^
**Commercial Cultivars**									
Kordia	26.42 ± 1.09 ^c^	9.81 ± 0.91 ^a^	0.66 ± 0.08 ^b,c^	2.69 ± 0.27 ^e^	0.29 ± 0.03 ^c^	0.73 ± 0.07 ^a^	0.43 ± 0.02 ^c^	93.01 ± 4.82 ^f^	2.37 ± 0.18 ^d,e^
Regina	31.93 ± 0.05 ^d^	12.38 ± 0.23 ^b,c^	0.89 ± 0.15 ^d^	2.77 ± 0.045 ^e^	0.26 ± 0.01 ^b,c^	0.96 ± 0.02 ^c,d^	0.63 ± 0.02 ^e^	74.19 ± 5.86 ^e^	2.47 ± 0.06 ^e^
Sylvia	79.59 ± 2.17 ^g^	10.19 ± 0.44 ^a^	0.73 ± 0.02 ^c^	1.95 ± 0.08 ^c^	0.18 ± 0.01 ^a^	1.04 ± 0.05 ^d,e^	0.28 ± 0.01 ^a^	90.00 ± 7.18 ^f^	2.28 ± 0.26 ^d,e^
local cultivars av.	30.00 ± 15.72 ^a^	11.66 ± 1.90 ^a^	0.58 ± 0.11 ^a^	1.45 ± 0.98 ^a^	0.35 ± 0.12 ^a^	1.30 ± 0.49 ^b^	0.39 ± 0.10 ^a^	27.12 ± 21.27 ^a^	1.81 ± 0.86 ^a^
commercial cultivars av.	45.98 ± 25.35 ^a^	10.79 ± 1.31 ^a^	0.76 ± 0.13 ^b^	2.47 ± 0.42 ^a^	0.25 ± 0.05 ^a^	0.91 ± 0.14 ^a^	0.45 ± 0.15 ^a^	85.73 ± 10.19 ^b^	2.37 ± 0.18 ^a^
***p*-value: group of cultivars**	N.S.	N.S.	0.009	N.S.	N.S.	0.031	N.S.	<0.0001	N.S.
***p*-value: cultivar**	<0.0001	0.0001	<0.0001	<0.0001	<0.0001	<0.0001	<0.0001	<0.0001	<0.0001

^1^ Data are presented as mean ± SD, with ANOVA *p*-values (*n* = 4); ^2^ Means in column followed by the same letter (^a–g^) are not significantly different at the 5% level of probability (*p* < 0.05) N.S.: not significant; N.D.: not detected.

**Table 6:** The content of carotenoids (in mg 100 g^-1^ FW) in sweet cherry fruit of local cultivars harvested in 2012 and 2013.

Sweet Cherry Cultivars	Lutein		Zeaxanthin		α*-*Carotene		β-Carotene	
	2012	2013	2012	2013	2012	2013	2012	2013
Altenburger Melonenkirsche	0.24 ± 0.02 ^a,1,2^	0.29 ± 0.01 ^a^	0.11 ± 0.00 ^a^	0.12 ± 0.00 ^a^	1.56 ± 0.02 ^a^	1.69 ± 0.02 ^a^	1.66 ± 0.03 ^a^	1.81 ± 0.04 ^a^
Bütners Rote Knorpelkirsche	0.32 ± 0.01 ^b^	0.41 ± 0.00 ^b^	0.12 ± 0.00 ^a^	0.14 ± 0.00 ^a^	1.71 ± 0.05 ^a^	1.87 ± 0.02 ^a^	1.77 ± 0.01 ^b^	1.90 ± 0.03 ^b^
Drogans Gelbe Knorpelkirsche	1.24 ± 0.01 ^c^	1.39 ± 0.02 ^c^	1.60 ± 0.02 ^b^	1.85 ± 0.03 ^b^	2.24 ± 0.01 ^b^	2.58 ± 0.04 ^b^	1.95 ± 0.05 ^c^	2.34 ± 0.02 ^c^
Elton Heart	2.32 ± 0.11 ^d^	2.73 ± 0.10 ^d^	1.87 ± 0.04 ^c^	2.12 ± 0.01 ^c^	2.88 ± 0.03 ^c^	3.05 ± 0.03 ^c^	2.03 ± 0.01 ^d^	2.88 ± 0.05 ^c^
local cultivars – av.	1.03 ± 0.84	1.21 ± 0.80	0.92 ± 0.81	1.06 ± 0.93	2.10 ± 0.52	2.30 ± 0.55	1.85 ± 0.15	2.23 ± 0.43
*p*-value	<0.0001	<0.0001	<0.0001	<0.0001	<0.0001	<0.0001	<0.0001	<0.0001

^1^ Data are presented as the mean ± SD, with ANOVA *p*-value (*n* = 4); ^2^ Means in the same columns followed by the same letter (^a–d^) are not significantly different at the 5% level of probability (*p* < 0.05).

**Table 7:** Correlations between antioxidant activity and polyphenols and β-carotene concentrations in fruits of local and commercial sweet cherry cultivars harvested in 2012 and 2013.

Sweet Cherry Cultivars		Polyphenols (mg 100g^−1^ FW)		β-Carotene (mg 100g^−1^ FW)	
		*r^2^* ^a^	*p*	*r^2^*	*p*
**Local Cultivars**	**Antioxidant Activity (mmol Trolox 100g^−1^ FW)**				
Altenburger Melonenkirsche		0.9017	0.0001	-	-
Bütners Rote Knorpelkirsche		0.7593	0.0022	-	-
Black from Turwia		0.8249	0.0007	-	-
Black Late		0.8400	0.0005	-	-
Drogans Gelbe Knorpelkirsche		0.8873	0.0001	0.7381	0.001
Elton Heart		0.8923	0.0001	0.8830	0.0010
**Commercial Cultivars**					
Kordia		0.9315	0.0001	-	-
Regina		0.8272	0.0007	-	-
Sylvia		0.8054	0.0010	-	-

^a^ Pearson’s coefficient (*n* = 8).
